# Negative Autogenous Control of the Master Type III Secretion System Regulator HrpL in *Pseudomonas syringae*

**DOI:** 10.1128/mBio.02273-16

**Published:** 2017-01-24

**Authors:** Christopher Waite, Jörg Schumacher, Milija Jovanovic, Mark Bennett, Martin Buck

**Affiliations:** Department of Life Sciences, Imperial College London, London, United Kingdom; University of California, Berkeley

## Abstract

The type III secretion system (T3SS) is a principal virulence determinant of the model bacterial plant pathogen *Pseudomonas syringae*. T3SS effector proteins inhibit plant defense signaling pathways in susceptible hosts and elicit evolved immunity in resistant plants. The extracytoplasmic function sigma factor HrpL coordinates the expression of most T3SS genes. Transcription of *hrpL* is dependent on sigma-54 and the codependent enhancer binding proteins HrpR and HrpS for *hrpL* promoter activation. *hrpL* is oriented adjacently to and divergently from the HrpL-dependent gene *hrpJ*, sharing an intergenic upstream regulatory region. We show that association of the RNA polymerase (RNAP)-HrpL complex with the *hrpJ* promoter element imposes negative autogenous control on *hrpL* transcription in *P. syringae* pv. *tomato* DC3000. The *hrpL* promoter was upregulated in a Δ*hrpL* mutant and was repressed by plasmid-borne *hrpL*. In a minimal *Escherichia coli* background, the activity of HrpL was sufficient to achieve repression of reconstituted *hrpL* transcription. This repression was relieved if both the HrpL DNA-binding function and the *hrp*-box sequence of the *hrpJ* promoter were compromised, implying dependence upon the *hrpJ* promoter. DNA-bound RNAP-HrpL entirely occluded the HrpRS and partially occluded the integration host factor (IHF) recognition elements of the *hrpL* promoter *in vitro*, implicating inhibition of DNA binding by these factors as a cause of negative autogenous control. A modest increase in the HrpL concentration caused hypersecretion of the HrpA1 pilus protein but intracellular accumulation of later T3SS substrates. We argue that negative feedback on HrpL activity fine-tunes expression of the T3SS regulon to minimize the elicitation of plant defenses.

## IMPORTANCE

The United Nations Food and Agriculture Organization has warned that agriculture will need to satisfy a 50% to 70% increase in global food demand if the human population reaches 9 billion by 2050 as predicted. However, diseases caused by microbial pathogens represent a major threat to food security, accounting for over 10% of estimated yield losses in staple wheat, rice, and maize crops. Understanding the decision-making strategies employed by pathogens to coordinate virulence and to evade plant defenses is vital for informing crop resistance traits and management strategies. Many plant-pathogenic bacteria utilize the needle-like T3SS to inject virulence factors into host plant cells to suppress defense signaling. *Pseudomonas syringae* is an economically and environmentally devastating plant pathogen. We propose that the master regulator of its entire T3SS gene set, HrpL, downregulates its own expression to minimize elicitation of plant defenses. Revealing such conserved regulatory strategies will inform future antivirulence strategies targeting plant pathogens.

## INTRODUCTION

Most agriculturally important bacterial plant pathogens utilize a type III secretion system (T3SS) as a channel for delivery of virulence proteins, known as effectors, into the plant cell cytoplasm (1). The T3SS consists of a conserved transmembrane base complex and a needle-like pilus appendage (2, 3). In plant pathogens, its early substrates, the harpins, function to form the translocon, a pore in the target cell membrane through which effectors are subsequently secreted. Effectors are structurally and functionally diverse (4), targeting key components of eukaryotic signaling pathways to suppress the two layers of plant immunity: (i) broadly acting innate defenses triggered by invariant pathogen-associated molecular patterns (PAMPs) and (ii) the rapid, localized, and pathogen-specific hypersensitive response (HR) triggered by evolved recognition of effectors (5).

Comprising over 50 disease-causing pathovars, many of which infect valuable crops such as tomato, bean, and rice, *Pseudomonas syringae* is the most highly developed model for T3SS-dependent plant pathogenesis and evolution of host specificity (6–8). *P. syringae* strains are found ubiquitously on leaf surfaces as well as in soil, freshwater, and precipitation. The *P. syringae* pv. *tomato* DC3000 pathovar (here DC3000) enters *Arabidopsis thaliana* leaves through wounds or stomata and replicates within the apoplast, causing chlorosis and necrotic lesions (8, 9).

The T3SS structural, helper, and regulatory proteins are encoded by a cluster of HR and conserved (*hrc*) and HR and pathogenicity (*hrp*) genes, flanked by variable effector loci within the Hrp pathogenicity island (10). In several plant pathogens, the extracytoplasmic function (ECF) sigma factor HrpL regulates the coordinated expression of the Hrp regulon via a conserved promoter motif, the *hrp-*box (11, 12). ECF sigma factors couple the expression of a functionally related gene set to perception of environmental cues (13). Transcription of *hrpL* is regulated by the alternative sigma-54 (σ^54^) factor, which requires activation by bacterial enhancer binding proteins (EBPs) bound at a distal promoter site called the upstream activation sequence (UAS) for transcription initiation (14). Integration host factor (IHF)-mediated DNA looping facilitates contact between the promoter-bound EBP complex, usually homohexameric, and the inactive RNA polymerase (RNAP)-σ^54^ complex. In *P. syringae*, transcription of *hrpL* is atypically activated by a heterohexamer comprising two codependent EBPs, HrpR and HrpS (15). Furthermore, HrpS is subjected to allosteric posttranslational inhibition by HrpV, which is in turn sequestered by HrpG (16).

Expression of the T3SS regulon is stimulated by minimal culture media mimicking the abiotic conditions of the leaf apoplast (17) and further enhanced by plant cells or soluble extracts (18). The importance of coordinated adjustments in gene expression for niche adaptation on the plant host is highlighted by the global changes in the *P. syringae* transcriptome evident upon transition from the leaf surface to the apoplast (19). However, how plant signals are perceived and transduced into the Hrp regulatory network remains poorly understood. Regulatory motifs such as feedback loops can influence the population-level behaviors of infecting pathogens. In particular, bistable expression of the T3SS drives heterogeneity and division of labor in both plant and animal pathogens (20, 21), including *P. syringae* (22). New insights into the regulatory networks underlying the T3SS and other bacterial virulence factors promise to inform strategies to manage plant disease. For example, by reducing the selection pressure for bacterial resistance, the use of antivirulence chemicals to “disarm” the T3SS by modulating its function or regulation represents an effective alternative to crop resistance breeding (23).

**FIG 1:**
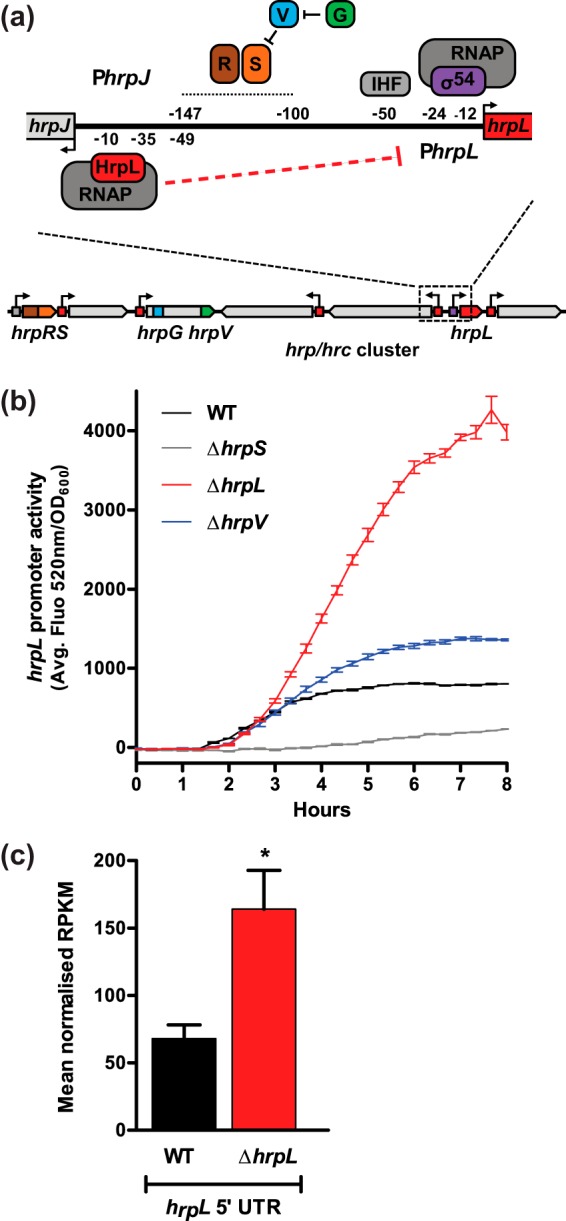
Negative feedback on *hrpL* transcription implied by the Δ*hrpL* mutant. (a) The regulation of *hrpL* transcription in *P. syringae*. The organization of the bidirectional promoter region between *hrpL* and *hrpJ*, including known binding sites for σ^54^, IHF, and HrpRS, is shown. HrpS activator function is regulated antagonistically by HrpV and HrpG. HrpL promotes transcription of *hrpJ* and other *hrp*-*hrc* operons via the *hrp*-box (red boxes). The study results propose a mechanism of negative autogenous control dependent on RNAP-HrpL binding at the *hrpJ* promoter (dashed red line). (b) *hrpL* promoter activity (fluorescence [Fluo]/OD_600_) in DC3000 wild-type (WT), Δ*hrpS*, Δ*hrpL*, and Δ*hrpV* strains carrying the pBBR1-P*hrpL*-*gfp* reporter plasmid under *hrp*-inducing conditions. Error bars represent standard errors of the means (SEM) of results of 3 biological replicates. (c) Transcription of chromosomal *hrpL* locus in wild-type and Δ*hrpL* strains inferred by RNA-seq. Data represent relative expression levels of a 5′ section of the *hrpL* transcript (−24 to +25 relative to ATG) under *hrp*-inducing conditions. The mean and quantile-normalized reads per kilobase per million (RPKM) values for two biological replicates per strain are shown with SEM. Differential expression is significant according to Baggerley’s test with false-discovery-rate (FDR) adjustment (*P* = 4.57 × 10^−6^).

The *hrpL* gene is transcribed divergently with respect to the HrpL-dependent *hrpJ* operon, sharing an intergenic upstream regulatory region in which the respective UAS and *hrp*-box elements are directly adjacent (Fig. 1a). *hrpJ* encodes a putative regulator of T3SS substrate preference (24), while the downstream *hrcV* and *hrcN* genes encode conserved subunits of the base complex. We examined the control of the *hrpL* and *hrpJ* promoters to test the hypothesis that regulatory interplay might exist between them. We show that *hrpL* expression is subject to negative autogenous control (*NAC*), mediated via HrpL binding at the *hrpJ* promoter. The DNA footprint of the RNAP-HrpL complex suggests that HrpL achieves repression by occluding the UAS- and IHF-binding sites of the *hrpL* promoter. Quantitative proteomics suggests that T3SS function is highly sensitive to the HrpL concentration, allowing us to propose possible physiological advantages of negative-feedback mechanisms in the context of the host plant.

## RESULTS

### HrpV-independent negative feedback on *hrpL* transcription.

The relative levels of activity of the *hrpL* promoter (P*hrpL*) across various regulatory mutant strains were compared using a transcriptional green fluorescent protein (GFP) fusion construct (pBBR1-P*hrpL*-*gfp*) encompassing the intergenic region shared between *hrpL* and *hrpJ* (Fig. 1a). Verifying the strict requirement of the HrpS coactivator for P*hrpL* activity under established *hrp*-inducing growth conditions, a basal level of GFP fluorescence, normalized for cell density, was observed in the Δ*hrpS* strain (Fig. 1b) (25). The striking 4-fold increase in fluorescence observed in the Δ*hrpL* strain in comparison to the wild-type (WT) strain after 8 h suggests that negative feedback acting on P*hrpL* in the wild-type strain had been relieved. Given that HrpV inhibits HrpS activity and that *hrpV* expression is directly dependent on the presence of HrpL, the downregulation of this regulator in the Δ*hrpL* strain partially accounts for the apparent negative feedback. Indeed, an increase in P*hrpL* activity in the Δ*hrpV* strain in comparison to wild type was observed. However, the fact that the upregulation of P*hrpL* activity apparent in the Δ*hrpL* strain was stronger than that in the Δ*hrpV* strain may suggest a novel HrpV-independent mechanism of negative feedback. The HrpL-mediated repression phenotype was verified at the level of the native transcript by transcriptome sequencing (RNA-seq), confirming that the differences in the levels of P*hrpL* activity observed were not an artifact of the reporter system. After 4 h under *hrp*-inducing conditions, while expression of the HrpL-dependent T3SS regulon was suppressed (see Data set S1 in the supplemental material), a 5′ section of the *hrpL* transcript (present in the Δ*hrpL* deletion construct) was upregulated in the Δ*hrpL* strain compared to the wild-type strain (Fig. 1c). The (+2.4-) fold change was approximately equivalent to the difference in the levels of reporter fluorescence observed at the same time point. Flow cytometry data confirmed that P*hrpL* activity conformed to approximately the normal distribution in both the wild-type and Δ*hrpL* strain populations (see Fig. S1a in the supplemental material). Moreover, broadly similar curves for optical density at 600 nm (OD_600_) confirmed that the differences in the levels of P*hrpL* activity across the strains tested were not artifacts of irregular cell growth (see Fig. S1b).

10.1128/mBio.02273-16.1 DATA SET S1  (Data 1) RNA-seq expression data for key T3SS genes in DC3000 wild-type and Δ*hrpL*. Statistically significant fold changes (FDR-adjusted *P* value < 0.05) are indicated in boldface characters. (Data 2) Peptide transitions analyzed by MRM-MS. Abbreviations: rep, replicate; ID, transition identifier; A, analyte; IS, internal standard; *, heavy amino acid residue. Download DATA SET S1, XLSX file, 0.02 MB. Copyright © 2017 Waite et al. 2017 Waite et al. This is an open-access article distributed under the terms of the Creative Commons Attribution 4.0 International license.

10.1128/mBio.02273-16.2 FIG S1  Effect of the Δ*hrpL* mutation on cell growth and the population distribution of *hrpL* promoter activity. (a) Histogram of GFP expression in populations of DC3000 cells after 8 h of growth in *hrp*-inducing medium. Triplicate populations of Δ*hrpL* (red shading) and wild-type (gray shading) strains were sampled for flow cytometry (10,000 events). Fluorescence signal is shown on a log_10_ scale. *hrpL* promoter activity followed a normal distribution across both the wild-type and Δ*hrpL* populations, with a higher average level of activity in the Δ*hrpL* strain. (b) Cell density (OD_600_) curves measured simultaneously with reporter fluorescence. Δ*hrpS*, Δ*hrpL*, and Δ*hrpV* gene deletions had a negligible effect on cell growth with respect to wild-type DC3000. Error bars represent SEM of results of 3 biological replicates. Download FIG S1, EPS file, 0.9 MB. Copyright © 2017 Waite et al. 2017 Waite et al. This is an open-access article distributed under the terms of the Creative Commons Attribution 4.0 International license.

### Negative feedback is dependent on HrpL concentration and DNA-binding function.

**FIG 2:**
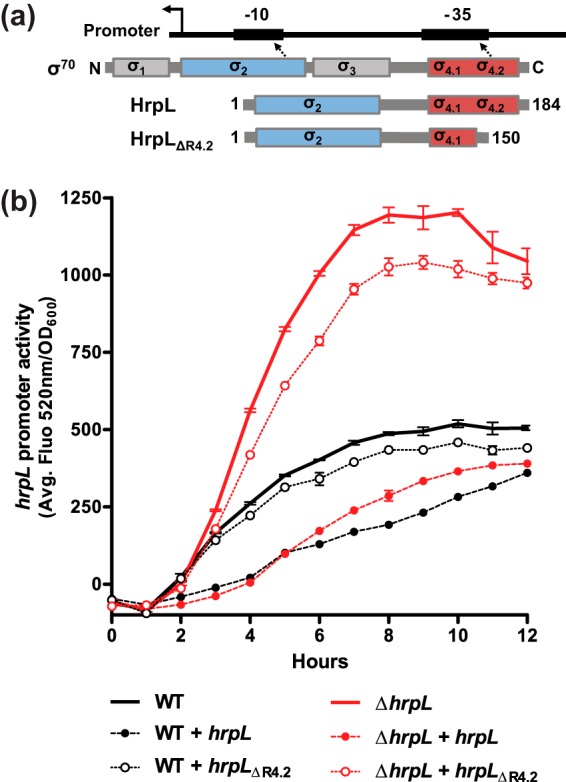
Negative feedback is dependent on the expression level and DNA-binding function of HrpL. (a) ECF σ factors retain the DNA-binding characteristics of the wider σ^70^-type family: region 2 (σ_2_) and region 4.2 (σ_4.2_) interact with the −10 and −35 promoter elements, respectively. The C-terminal truncation mutant, HrpL_ΔR4.2_, lacks conserved −35 element binding determinants. (b) *hrpL* promoter activity in DC3000 wild-type and Δ*hrpL* strains carrying the pBBR1-P*hrpL*-*gfp* reporter and plasmid-encoded *hrpL* variants under *hrp*-inducing conditions. HrpL (filled circles) and HrpL_ΔR4.2_ (hollow circles) were expressed from the pSEVA224-*hrpL* and pSEVA224-*hrpL*_ΔR4_._2_ plasmids, respectively, while empty pSEVA224 (bold line) was used to control for plasmid load. Error bars represent SEM of results of 3 biological replicates.

To examine whether HrpL-dependent negative feedback requires its canonical sigma factor function, the Δ*hrpL* strain was complemented with a variant of HrpL impaired in DNA binding. ECF sigma factors are strongly dependent on the C-terminal 4.2 region for interaction with promoter DNA at the −35 element (26) (Fig. 2a). The strong similarity between the predicted HrpL and known *Escherichia coli* σ^E^ protein structures (data not shown) was used to infer the location of the HrpL region 4.2. A truncated HrpL variant (positions 1 to 150; HrpL_ΔR4.2_) was generated that was able to bind core RNA polymerase (see Fig. S2a and S2b) but unable to activate HrpL-dependent transcription (see Fig. S3a and S2c). In both the Δ*hrpL* strain and the wild-type strain, full-length HrpL hyperrepressed P*hrpL* activity when expressed from the pSEVA224 plasmid (Fig. 2b), suggesting that the intensity of negative feedback is dependent on the HrpL concentration. In contrast, the HrpL_ΔR4.2_ DNA-binding mutant repressed P*hrpL* activity only weakly, confirming that negative feedback by HrpL is primarily dependent on promoter binding rather than an alternative function. This disqualifies DNA-independent competition between HrpL and σ^54^ for free RNAP molecules as a sole explanation for the observed differences in P*hrpL* activity.

10.1128/mBio.02273-16.3 FIG S2  *In vitro* abortive transcription activity of copurified RNAP-HrpL and RNAP-HrpL_ΔR4.2_ complexes on *hrpJ* promoter probes. (a) RNAp core (6His-β′) was coexpressed in *E. coli* with HrpL or HrpL_ΔR4.2_. Total protein was sampled following cell disruption (Whole cell) and purification by Ni-affinity chromatography (Purified) and compared alongside size markers (L) by staining of SDS-PAGE gels with Coomassie dye. The following components are signified by arrows: RNAP α subunit (34.9 kDa); HrpL (22.6 kDa); HrpL_ΔR4.2_ (18.9 kDa); RNAP ω subunit (10.5 kDa). (b) Detection of HrpL variants by Western blotting targeting a C-terminal Myc tag. Proteins were transferred directly from the gel shown in panel a to a polyvinylidene difluoride (PVDF) membrane and incubated with an anti-Myc antibody. (c) Abortive initiation on supercoiled P*hrpJ* or P*hrpJ*(Δ35e) promoter DNA was assayed in the presence of RNAP core enzyme or RNAP-HrpL or RNAP-HrpL_ΔR4.2_ holoenzymes. An abortive small priming RNA (spRNA) product (predicted CpApUpApApU) was separated from free nucleotides by electrophoresis. The average level of initiation activity across 3 replicates is given as a percentage relative to RNAP-HrpL on wild-type promoter DNA. Initiation was inhibited >90% in the presence of RNAP-HrpL_ΔR4.2_ and/or P*hrpJ*(Δ35e). Download FIG S2, EPS file, 7.9 MB. Copyright © 2017 Waite et al. 2017 Waite et al. This is an open-access article distributed under the terms of the Creative Commons Attribution 4.0 International license.

10.1128/mBio.02273-16.4 FIG S3  Plasmid-borne expression of HrpL and HrpL_ΔR4.2_ in DC3000. (a) HrpL dependence of *hrpJ* promoter fusion activity in DC3000. *hrpJ* promoter activity in wild-type (black) and Δ*hrpL* (red) strains carrying both pBBR1-*rfp*-P*hrpL*-*gfp* and plasmids for heterologous expression of *hrpL* under *hrp*-inducing conditions is reported. *hrpL* (full circles) or *hrpL*_ΔR4.2_ (empty circles) genes were expressed from the pSEVA224 plasmid. Empty pSEVA224 vector (bold lines) was used as a plasmid load control. Autofluorescence by DC3000 in the absence of reporter was also measured (gray dashed line). Error bars represent SEM of results of 3 biological replicates. *hrpJ* promoter activity was negligible, with respect to autofluorescence, in the Δ*hrpL* strain, confirming the HrpL dependence of transcription. Complementation with plasmid-borne *hrpL*, but not inactive *hrpL*_ΔR4.2_, resulted in strong upregulation of P*hrpJ* with respect to wild-type results. (b) Cell density (OD_600_) curves measured simultaneously with reporter fluorescence. *hrpL* and *hrpL*_ΔR4.2_ overexpressed from pSEVA224 had a negligible effect on the cell growth of wild-type DC3000, with respect to an empty vector control for plasmid load. Error bars represent SEM of results of 3 biological replicates. Download FIG S3, EPS file, 1.1 MB. Copyright © 2017 Waite et al. 2017 Waite et al. This is an open-access article distributed under the terms of the Creative Commons Attribution 4.0 International license.

### HrpL is sufficient for autogenous negative control.

**FIG 3:**
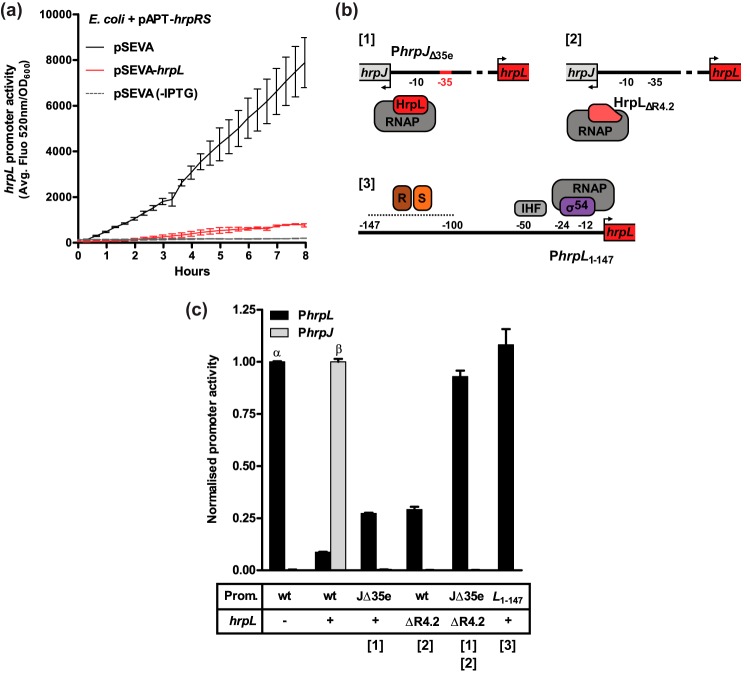
(a) *hrpL* promoter activity in an *E. coli* s17λpir strain carrying the pBBR1-P*hrpL*-*gfp* reporter in LB medium. Heterologous expression of *hrpL* from pSEVA614-*hrpL* (red) and *hrpRS* from pAPT-*hrpRS* was induced with 0.1 mM IPTG. The empty pSEVA614 vector (black) was used to control for plasmid load. HrpRS-independent P*hrpL* activity was measured in the absence of IPTG (dashed gray line). Error bars represent SEM of results of 3 biological replicates. (b) Mutations disrupting the association of the RNAP-HrpL with P*hrpJ* DNA. [1] Substitution of the *hrp*-box −35 element (GGAACT > AACACT). [2] Truncation of HrpL DNA-binding region 4.2. [3] Exclusion of P*hrpJ* using a minimal promoter sequence sufficient for P*hrpL* activity (−147 to +1). (c) The relative activity levels of P*hrpL* and P*hrpJ* seen when HrpL DNA-binding function was disrupted. The expression states of *hrpL* and the *cis*- and *trans*-acting mutations acting under each condition are tabulated below the graph. P*hrpL* (GFP) and P*hrpJ* (RFP) activities were measured simultaneously under all conditions using the reporters pBBR1-*rfp*-P*hrpL*-*gfp* (wt) or pBBR1-*rfp*-P*hrpL*(Δ35e)-*gfp* (JΔ35e), with the exception of the minimal pBBR1-P*hrpL*(147)-*gfp* reporter (L_1-147_; far-right column). *hrpL* (+) or *hrpL*_ΔR4_._2_ (ΔR4.2) were expressed from the pSEVA614 plasmid, the empty vector being used as a proxy for the Δ*hrpL* condition (−). HrpRS proteins were expressed from pAPT-hrpRS under all conditions. Promoter (Prom.) activities given are normalized relative to the maximum activity state for each condition after 8 h of growth in LB as follows: for P*hrpL*, empty pSEVA614/wild-type promoter (α); for P*hrpJ*, pSEVA614-*hrpL*/wild-type promoter (β). Error bars represent SEM of results of 3 biological replicates.

*E. coli* has been utilized previously as a heterologous model system in which to study the initiation of *hrpL* transcription independently of the wider DC3000-specific regulatory network (15). In this study, a multiple plasmid-based system was engineered in the *E. coli* s17λpir strain with IPTG (isopropyl-β-d-thiogalactopyranoside)-induced heterologous expression of *hrpRS* from pAPT-*hrpRS*, driving activation of the pBBR1-P*hrpL*-*gfp* reporter. Negligible fluorescence was observed in the absence of IPTG (Fig. 3a). In this *E. coli* system, the additional effect of HrpL expression was studied by introducing the pSEVA614-*hrpL* plasmid, also induced via the use of IPTG. Compared to the results seen with the empty pSEVA614 vector control, HrpL expression strongly repressed P*hrpL* activity (by approximately 10-fold) after 8 h. Heterologous *hrpL* expression did not alter the levels of cell growth (see Fig. S3b), negating the possibility of pleiotropic effects on cell physiology. Furthermore, the results of both a transcriptional fusion assay and analysis of transcript levels indicated that HrpL does not significantly influence HrpRS expression (see Fig. S4 and Data set S1). These observations together suggest that HrpL alone, rather than a factor in the DC3000 HrpL-dependent regulon, is sufficient to autogenously repress P*hrpL* activity.

10.1128/mBio.02273-16.5 FIG S4  Activity of the *hrpRS* promoter in Δ*hrpL*. *hrpRS* promoter activity reported in WT (black) and Δ*hrpL* (red) strains carrying pBBR1-P*hrpRS*-*gfp* under *hrp*-inducing conditions. Error bars represent SEM of results of 3 biological replicates. No significant difference in *hrpRS* promoter activity is evident in the Δ*hrpL* strain with respect to wild-type DC3000 results during 8 h in *hrp*-inducing medium. Download FIG S4, EPS file, 0.5 MB. Copyright © 2017 Waite et al. 2017 Waite et al. This is an open-access article distributed under the terms of the Creative Commons Attribution 4.0 International license.

### Negative autogenous control is dependent on the adjacent *hrpJ* promoter.

The *E. coli* test system was developed further in order to test the hypothesis that HrpL exerts *NAC* as a result of σ factor function at the adjacent *hrpJ* promoter. pBBR1-P*hrpL*-*gfp* was modified to generate a bidirectional and dual-color reporter, pBBR1-*rfp*-P*hrpL*-*gfp*, in which the P*hrpL* and P*hrpJ* promoter elements are fused to GFP and red fluorescent protein (RFP), respectively, enabling their relative levels of activity to be measured simultaneously. A second reporter was derived in which the *hrp*-box −35 element at P*hrpJ* was disrupted by site-directed mutagenesis. Substitution of any nucleotide in the *hrp*-box −35 element (GGAAC) abolishes HrpL function (12). Therefore, a trinucleotide GGA>AAC substitution was introduced in the pBBR1-*rfp*-P*hrpL*(Δ35e)-g*fp* reporter to inhibit the association of HrpL at this site. Finally, a further reporter construct was generated consisting of the minimal sufficient P*hrpL* promoter sequence (−147 to +1) (15) but lacking the entire P*hrpJ* promoter, pBBR1-P*hrpL*(147)-*gfp*. Together with the pSEVA614-*hrpL* and pSEVA614-*hrpL*_Δ4.2_ expression plasmids, this series of reporters were used to investigate the specific role of HrpL DNA-binding function at P*hrpJ* for *NAC* (Fig. 3b). P*hrpJ* activity was observed in *E. coli* but only in the presence of full-length HrpL and an intact promoter sequence (here, the unmodified condition) (Fig. 3c). Both the *hrp*-box and HrpL_ΔR4.2_ mutations abolished P*hrpJ* activity, inferred by negligible RFP fluorescence and inhibition of *in vitro* transcription from P*hrpJ* by >90% (see Fig. S2c). Compared to the results seen under the unmodified condition, in which *NAC* was apparent, both mutations induced a modest increase in P*hrpL* activity, corresponding to approximately 25% of that observed in the absence of HrpL. When the two mutations were combined in the same strain, maximum P*hrpL* activity was almost completely restored (93%). These data suggest that disrupting the ability of HrpL to associate with the P*hrpJ hrp*-box relieves *NAC* for P*hrpL*. The inability of either mutation alone to completely derepress P*hrpL* activity is suggestive of residual interactions between the RNAP-HrpL holoenzyme complex and promoter that are sufficient to impose partial *NAC* but unable to initiate transcription at P*hrpJ*. However, in a HrpL_ΔR4.2_ background, the fact that the addition of the *hrp-*box mutation relieves *NAC* is sequence-specific evidence for a mechanism involving P*hrpJ*. Indeed, *NAC* was completely relieved in the absence of the entire P*hrpJ* element [pBBR1-P*hrpL*(147)-*gfp*].

### The P*hrpJ*-bound RNAP-HrpL complex partially occludes the *hrpL* promoter.

**FIG 4:**
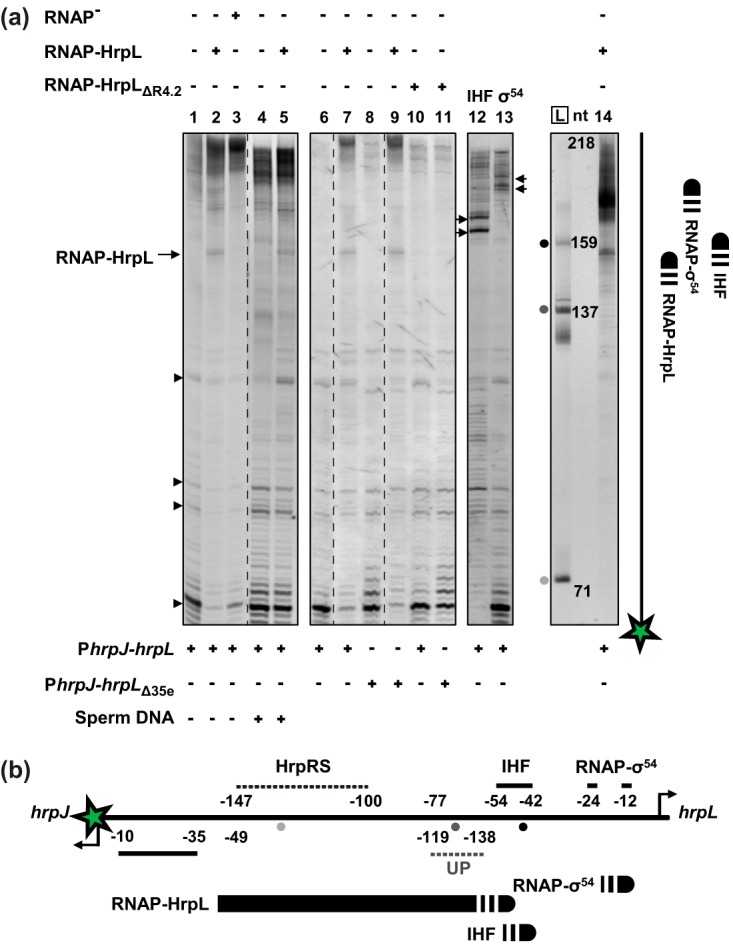
Exonuclease III footprint of RNAP-HrpL holoenzyme on *hrpJ*-*hrpL* promoter DNA. (a) P*hrpJ-hrpL* and P*hrpJ-hrpL*_Δ35e_ promoter probes labeled with Cy3 dye at the P*hrpJ* terminus (green star) were incubated at 200 nM with protein complexes prior to incomplete 3′ to 5′ digestion by ExoIII and separation of the fragments in an 8% polyacrylamide gel. Proteins were analyzed at the following concentrations: RNAP-HrpL and RNAP-HrpL_ΔR4.2_, 5 μM; RNAP core enzyme, 4 μM; IHF, 1 μM; RNAP-σ^54^, 400 nM and 2.4 μM. Salmon sperm DNA (1 μM) was used as nonspecific competitor DNA. Sites of 3′ digestion inhibition by DNA-bound complexes of RNAP-HrpL, IHF, and RNAP-σ^54^ are annotated (arrows, lanes 12 and 13) and summarized (far-right cartoon). Multiple independent gels (differentiated by boxes) are aligned with reference to consistent digestion fragments (arrowheads). Dashed lines signify exclusion of gel lanes for clarity. Size markers of lengths of 71, 137, and 159 nt (L) are shown alongside the RNAP-HrpL footprint (lane 14). (b) A schematic representation of the 3′ boundaries of DNA-bound RNAP-HrpL, IHF, and RNAP-σ^54^ complexes in the context of known regulatory elements of the P*hrpJ-hrpL* promoter region, annotated relative to the respective transcription start sites. The relative locations of the three size markers electrophoresed alongside footprint products, as annotated in panel a, are signified by shaded circles.

The interaction between HrpL and the promoter DNA shared between *hrpL* and *hrpJ* was further characterized *in vitro*. HrpL readily formed insoluble inclusion bodies when overexpressed for protein purification, here (data not shown) and in previous studies (12, 27). Therefore, the solubility of HrpL and HrpL_ΔR4.2_ was maintained via copurification in complex with an *E. coli* RNAP with a His tag at the β subunit (28). The purified RNAP-HrpL holoenzyme activated transcription from P*hrpJ in vitro*, confirming its ability to both bind promoter DNA and form an open promoter complex (see Fig. S2c). However, the RNAP-HrpL_ΔR4.2_ mutant achieved approximately 4% of the wild-type activity. Exonuclease III (ExoIII) footprinting was performed on DNA probes comprising the entire P*hrpJ*-*hrpL* intergenic region, labeled at the P*hrpJ* terminus such that the 3′ to 5′ directionality of ExoIII might reveal the distal boundary of the RNAP-HrpL complex. The HrpL-RNAP complex blocked ExoIII digestion on both P*hrpJ-hrpL* and P*hrpJ-hrpL*_Δ35e_ promoter probes and in the presence of nonspecific competitor DNA (Fig. 4a). However, both the RNAP-HrpL_ΔR4.2_ holoenzyme and the RNAP core enzyme (no bound σ factor) failed to produce an equivalent footprint, suggesting that the ExoIII block is specific to the interaction between HrpL and promoter DNA. The fact that the footprint was observed on both promoter probes supports the conclusion that HrpL maintains some affinity for the mutated Δ35e *hrp*-box sequence, as suggested by the presence of residual *NAC in vivo* (Fig. 3c). In the context of DNA size markers and the IHF and RNAP-σ^54^ footprints, RNAP-HrpL blocked ExoIII digestion at a position within the IHF consensus recognition sequence of P*hrpL* (Fig. 4b). This implies that P*hrpJ*-bound RNAP-HrpL occludes a significant region of P*hrpL*, including the predicted UAS for HrpRS binding (15) and part of the IHF recognition sequence. *NAC* might therefore be manifest via interference with HrpRS and/or IHF DNA binding by RNAP-HrpL.

### Misregulation of *hrpL* inhibits T3SS function in culture.

**FIG 5:**
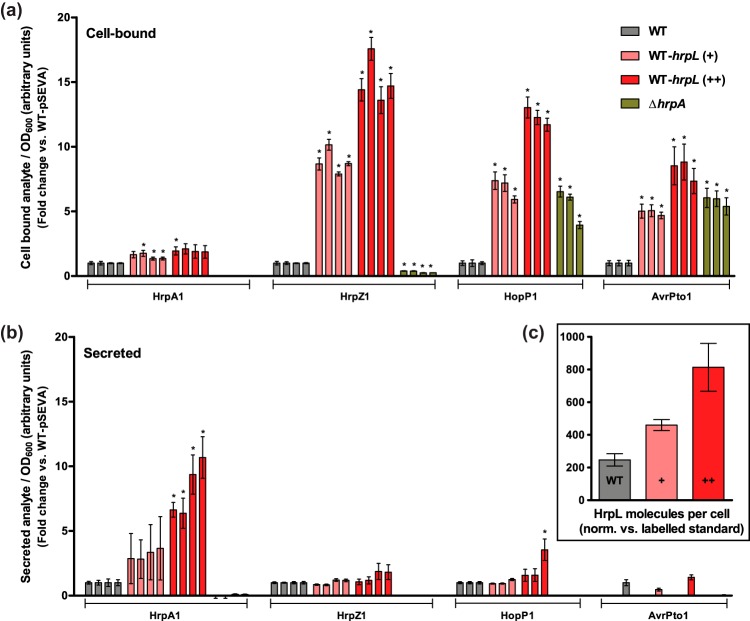
The effect of *hrpL* misregulation on expression and secretion of T3SS proteins. (a and b) Cell-bound (cell pellet) (a) and secreted (supernatant) (b) protein fractions were extracted from DC3000 cell cultures after 24 h in *hrp*-inducing medium for targeted protein quantification via LC-MRM-MS. The pSEVA224-33-*hrpL* (+) and pSEVA224-31-*hrpL* (++) plasmids were used to overexpress *hrpL*. An empty pSEVA224 vector was used as a plasmid load control. The Δ*hrpA* strain was used as a control for T3SS-independent release of protein. Multiple peptide transitions per protein were analyzed for calculation of relative target protein abundances between strains. From left to right, the columns shown represent the following transitions (see Data Set S1): for HrpA1, ISATa, ISATb, LTNLa, and LTNLb; for HrpZ1, AQFPa, AQFPb, SANSa, and SANSb; for HopP1, GQLNa, GQLNb, and NSNS; and for AvrPto1, HQLAa, HQLAb, and VSNN. The analyte transition peak intensities were normalized against sample cell density (OD_600_) and subsequently the wild-type (WT) pSEVA224 peak intensity to give data for fold change between strains. Absent column bars indicate that transitions were undetectable. (c) The absolute HrpL copy number in each intracellular sample was calculated using the ratio between the sample peptide (QPSS) and heavy-isotope standard peptide (QPSS-IS) transition peak intensities, normalizing for OD_600_ and standard concentration. Protein copy number data assume an OD_600_ of 1.0 = 10^9^ CFU ml^−1^. Error bars represent SEM of results of 3 biological replicates.

To infer the effect of *NAC* on T3SS function, the sensitivity of T3SS protein secretion to variations in HrpL copy numbers was investigated using targeted protein mass spectrometry (MS). We have previously developed a method for quantitative analysis of T3SS proteins secreted by DC3000 into *hrp*-inducing culture medium (HIM) (29). Briefly, shotgun MS was utilized to identify extracellular T3SS-associated proteins, prior to the most readily detectable signature peptide/fragment ion pairs (transitions) being selected for targeted and highly sensitive quantitation using multiple-reaction monitoring (MRM)-MS (30). Protein extract was “spiked” with a known concentration of heavy isotope-labeled standard such that absolute quantification of a specific target could be achieved upon ratiometric comparison of mass-distinguishable sample and standard transition peaks. In this study, the relative intracellular and extracellular abundances of four key T3SS-associated proteins (the pilus subunit HrpA1, the harpins HrpZ1 and HopP1, and the effector AvrPto1) were analyzed in a HrpL-concentration-dependent manner, using multiple peptide transitions for increased robustness. Given that HrpJ is a regulator of secretion (24), mutations that relieve *NAC* via disruption of P*hrpJ* function were considered unsuitable for assays of T3SS activity. Instead, HrpL was overexpressed from the pSEVA224-31-*hrpL* and pSEVA224-33-*hrpL* plasmids, using synthetic ribosome binding sites (strong and weak, respectively) to specify expression levels. Cell cultures were maintained for an extended 24 h under *hrp*-inducing conditions to enable accumulation of detectable extracellular protein before cell-bound and secreted protein fractions were extracted. The four T3SS-associated proteins were found in all supernatant samples with the exception of the Δ*hrpA1* strain, confirming that their release was dependent on the presence of the T3SS pilus and was not an artifact of cell lysis. A heavy-isotope-labeled standard was used for the quantification of intracellular HrpL copy number (Fig. 5c). Approximately 200 to 250 copies of HrpL were detected in the wild-type cell. The addition of the pSEVA224-33-*hrpL* or pSEVA224-31-*hrpL* plasmid increased the HrpL copy number to approximately 450 or 800, respectively. The effect of the HrpL concentration on the abundance of T3SS-associated proteins was more significant in the intracellular fractions (Fig. 5a). A 3-fold increase in the HrpL concentration (pSEVA224-31-*hrpL* versus pSEVA224) resulted in 15-, 12-, and 8-fold increases in HrpZ1, HopP1, and AvrPto1 abundance, respectively. Similarly, a 2-fold increase in HrpL (pSEVA224-33-*hrpL* versus pSEVA224) resulted in a 5-fold or greater increase in the abundance of these proteins. The HrpL concentration had no effect on the intracellular abundance of the housekeeping protein σ^70^ (RpoD) or Lon protease (see Fig. S5), implying that its overexpression has negligible pleiotropic effects on protein synthesis and the wider proteome. In the secreted fraction, only the abundance of the HrpA1 pilus protein increased in correlation with the HrpL concentration (Fig. 5b). In contrast, with the exception of a single HopP1 transition (NSNS), there was no significant difference in the levels of extracellular abundance of HrpZ1, HopP1, or AvrPto1. These data suggest that an increase in the HrpL copy number results in an intracellular accumulation of harpins and effectors but not an increase in their secretion rate. Accumulation can be explained by increased T3SS protein expression or substrate saturation of the T3SS or both. HrpL-dependent overexpression of the T3SS regulon is implied both by the correlation between HrpA1 translocation rate and HrpL copy number and by the increase in P*hrpJ* activity observed upon addition of plasmid-borne *hrpL* (see Fig. S3a). However, given that a HrpL-dependent increase in secretion was observed only for HrpA1, the increase in intracellular abundance of the other T3SS substrates suggests that there was a limitation in translocation rate and that their wild-type abundance was nearly saturating. We propose that *NAC* by HrpL does not significantly downregulate T3SS activity under wild-type conditions but rather acts to prevent expression of surplus substrates. Supporting this hypothesis, an increased HrpL expression rate had a negligible effect on DC3000 fitness during infection of host *Arabidopsis thaliana* seedlings (see Fig. S6).

10.1128/mBio.02273-16.6 FIG S5  (a) RpoD and (b) Lon protease abundance in intracellular protein fractions. Total cell-bound (cell pellet) protein was extracted from DC3000 cell cultures after 24 h in *hrp*-inducing medium for targeted protein quantification via LC-MRM-MS. The pSEVA224-33-*hrpL* (+) and pSEVA224-31-*hrpL* (++) plasmids were used to overexpress *hrpL*. Empty pSEVA224 was used as a plasmid load control in the wild-type strain. From left to right, the column bars shown represent the transitions analyzed for relative protein abundance (see Data Set S^1^) as follows: for RpoD, FGAVa, FGAVb, IPVHa, and IPVHb; for Lon, TSLAa and TSLAb. The analyte transition signal intensities were normalized against sample cell density (OD_600_). Error bars represent SEM of results of 3 biological replicates. Download FIG S5, EPS file, 0.7 MB. Copyright © 2017 Waite et al. 2017 Waite et al. This is an open-access article distributed under the terms of the Creative Commons Attribution 4.0 International license.

10.1128/mBio.02273-16.7 FIG S6  *Arabidopsis thaliana* seedling infection assays. (a) Col-0 seedlings were flood inoculated with bacterial suspensions of DC3000 wild-type and Δ*hrpL* strains at a cell density of 5 × 10^5^ CFU ml^−1^. Three replicate groups of 16 seedlings were flooded with each strain. Mock inoculations were performed with 10 mM MgCl_2_ solution as a control. Bacterial populations were quantified at 0, 1, 2, and 3 days postinfection (dpi). Error bars represent SEM for 3 samples, each consisting of 3 seedlings. Asterisks denote statistical significance in population size with respect to wild-type control results, based on a two-tailed *t* test. (b) Disease phenotypes of groups of three representative seedlings photographed 3 days postinfection. Symptoms, including chlorosis and lesion formation, are visible after infection with the wild-type strain but not after infection with the Δ*hrpL* strain or mock infection. (c) The effect of graded HrpL overexpression on *in planta* fitness. Seedlings were flood inoculated with DC3000 bacterial suspensions as described for panel a. The growth of wild-type populations carrying a series of pBBR1MCS4 plasmids for *hrpL* (plain bars) overexpression was monitored over 3 dpi. *hrpL* expression was regulated by synthetic ribosome binding sites (RBSs) (activity of pSEVA224-31-*hrpL* [31] > activity of pSEVA224-33-*hrpL* [33]). The pBBR1MCS4 vector was used as a plasmid control (black). *hrpL*_ΔR4.2_ (hashed bars) was similarly overexpressed as a control for sigma-independent effects of HrpL. Neither HrpL overexpression nor HrpL_ΔR4.2_ overexpression had any significant effect on *in planta* fitness with respect to plasmid control results (two-tailed *t* test). Download FIG S6, EPS file, 16.4 MB. Copyright © 2017 Waite et al. 2017 Waite et al. This is an open-access article distributed under the terms of the Creative Commons Attribution 4.0 International license.

## DISCUSSION

Feedback control mechanisms are utilized widely in mechanical and electrical systems to allow optimal performance to be maintained autogenously. Analogous mechanisms have evolved in a variety of biological systems, from gene networks to predator-prey cycles, and impart robustness to molecular or environmental fluctuations. Feedback loops represent common motifs in bacterial genetic circuits, which they endow with complex regulatory dynamics such as switching and oscillation. Around 40% of transcription factors in *E. coli* are subject to negative autogenous control (*NAC*) (31). Side-by-side comparisons of synthetic gene circuits have shown that the advantages of this particular mechanism can include robustness against gene expression noise, rapid response time, and population-wide homogeneity (32, 33). We now show that HrpL, the master regulator of T3SS expression in DC3000, exerts *NAC* of *hrpL* transcription as a direct result of its canonical σ-factor function at the adjacent *hrpJ* promoter, thus validating the hypothesis that the close synteny of these two genes imposes novel regulatory coupling between them. Independent lines of evidence, from both a plasmid-borne reporter fusion and the transcriptome, suggest that P*hrpL* is subject to negative feedback, relieved by the Δ*hrpL* deletion. Furthermore, heterologous reconstitution of *hrpL* transcription in *E. coli*, independently of the wider DC3000-specific regulatory network, reveals that a significant component of negative feedback by HrpL is both autogenous and dependent on the *hrpJ* promoter element.

### Negative autogenous control of a σ^54^-depdendent promoter.

The *hrp*-box at P*hrpJ* is situated only 15 nucleotides (nt) upstream of the putative UAS site proposed by Jovanovic et al. (15). Extrinsic *cis*-regulatory elements can repress σ^54^-dependent transcription initiation by altering local DNA geometry (34, 35) or interfering with DNA binding of either the activator complex (36) or σ^54^ itself (37). The example most analogous to the control of *hrpL* described here is the regulation of the divergent *atrZ*-*atzDEF* promoter region in *Pseudomonas* sp. strain ADP. Upon binding at a single recognition site, the LysR-type transcription factor AtrZ both activates *atzDEF* expression and represses σ^54^-dependent transcription of *atrZ* (37, 38). However, a notable contrast with *hrpL* is that activation of *atrZ* transcription by NtrC occurs independently of a UAS.

The *in vivo* analyses presented suggest that the mechanism of *NAC* by HrpL is dependent on DNA binding at P*hrpJ* but not subsequent transcription initiation. The torque generated by transcription leads to negative supercoiling in upstream DNA which can be sufficient to alter neighboring gene expression (39). However, the fact that *NAC* is maintained despite the elimination of P*hrpJ* activity due to either the *hrp*-box (Δ35e) or HrpL_ΔR4.2_ mutation alone negates torsional stress as its primary mechanism. The requirement for the two mutations to be acting in parallel for complete relief of repression implies that residual and potentially low-affinity interactions between the RNAP-HrpL holoenzyme and promoter DNA are sufficient to impose autogenous control. In support of this idea, the Δ35e mutation does not destabilize the RNAP-HrpL footprint on promoter DNA, corroborating previous evidence that HrpL maintains binding affinity for the *hrp*-box in spite of substitutions that inactivate transcription (12). Furthermore, the necessity of the Δ35e mutation for complete relief of *NAC* confirms that its mechanism is DNA sequence specific rather than acting from solution, for example, via competition between σ factors for RNAP.

As a classical ECF σ factor, the RNAP-σ^E^ holoenzyme occupies heat shock promoter DNA up to position −60 (40). If this DNA-binding property is common to the members of the ECF family, then it might be predicted *a priori* that P*hrpJ*-bound RNAP-HrpL overlaps the distal region of the P*hrpL* UAS. Surprisingly, the ExoIII footprinting data presented here suggest that this holoenzyme occupies promoter DNA further than 130 nt upstream of the transcription site, occluding completely the UAS and partially the IHF recognition sequence of P*hrpL*. One possible explanation relates to the function of the C-terminal domain of the RNAP α subunit (α-CTD) which, separated from the RNAP core enzyme by a flexible linker, can associate with distal promoter elements upstream of the σ recognition sequence (41). The regulation of the *E. coli lac* operon by the cyclic AMP receptor protein (CRP) requires an interaction with the α-CTD which can occur at the −92 position (42). The α-CTD also interacts with AT-rich upstream (UP) elements independently of protein interaction partners (43). Interestingly, a sequence resembling the UP element consensus sequence (43) is present upstream of P*hrpJ*, close to the location of the RNAP-HrpL footprint (Fig. 4b and Fig. S7 in the supplemental material). It is therefore plausible that the α-CTD is mediating *NAC* via an interaction with this putative UP element, although further work is required to verify this hypothesis. Given the location of the footprint, the most likely mechanisms of repression include inhibition of (i) HrpRS binding, (ii) IHF binding, or (iii) IHF-dependent DNA looping.

10.1128/mBio.02273-16.8 FIG S7  Putative UP element sequence upstream of the *hrpJ* promoter. (a) Position of putative UP element (UP J) with respect to the IHF recognition sequence and σ^54^ promoter of the P*hrpL*. (a) Alignment of UP J and known functional UP elements (*rrnB1* P1, *rrnD1* P1) with the consensus UP element (Estrem et al. [43]). Conserved positions are indicated in boldface and underlined characters. W, A or T nucleotide; N, any nucleotide. Download FIG S7, EPS file, 0.7 MB. Copyright © 2017 Waite et al. 2017 Waite et al. This is an open-access article distributed under the terms of the Creative Commons Attribution 4.0 International license.

Examples demonstrating direct repression of σ^54^-dependent transcription are rare because strict activator-dependent initiation usually negates the need for additional control. Therefore, the fact that multiple feedback mechanisms converge during *hrpL* transcription highlights the importance of fine-tuning T3SS expression in DC3000. In addition to *NAC* by HrpL, described here, HrpS activity is negatively regulated by HrpV binding (16) and *hrpRS* transcription is believed to be positively regulated via HrpA1, albeit by an unknown mechanism (44, 45). The fact that negative feedback on *hrpL* expression is partially autogenous represents an interesting contrast with the elegant negative-feedback system that couples T3SS expression to injectisome assembly in *Pseudomonas aeruginosa* (46). The AraC-type master regulator of T3SS gene expression, ExsA, is posttranslationally regulated by a series of antiactivators, which sequester one another in turn. Once the T3SS secretion channel is open, the export of ExsE triggers a signaling cascade, which ultimately liberates ExsA for upregulation of T3SS gene expression. There is no evidence to suggest that HrpL is regulated by an anti-σ factor, as is common among the ECF family, but it is plausible that the reported positive-feedback mechanism mediated by HrpA1 is by definition coupled to pilus function.

### The physiological significance of negative feedback by HrpL.

Given that the T3SS is a key determinant of *P. syringae* pathogenicity, it is assumed that any mechanism inhibiting its expression must impart a net positive fitness effect or be subject to negative selection. Singh and Hespanha propose that the potential fitness cost of decreased gene expression due to *NAC* can be outweighed if the gene in question is environmentally induced, if it functions at a low protein copy number, and if stochastic transitions between threshold expression states are unfavorable (47). Our current understanding of HrpL expression suggests that it fits these criteria closely. This study has shown that HrpL functions at relatively low abundance, as is generally the case for ECF family σ factors (48). Moreover, given that it responds to both the metabolic state (49) and plant-derived signals (18), the probability is high that considerable extrinsic noise is associated with *hrpL* expression. Finally, it can be assumed that stochastic inactivation of T3SS expression during an established interaction with the plant cell is deleterious.

Limitation of T3SS expression may be advantageous in the context of *P. syringae* ecology. A side effect of strong host specificity, defined by the existence of highly evolved effector protein repertoires, is susceptibility to the adaptive immune responses of non-host-plant species. Many *P. syringae* pathovars can survive asymptomatically on species outside their host range (9, 50). Thus, given that T3SS expression is broadly induced by cell-free exudates of both host and nonhost plants (18), it is plausible that there is a fitness trade-off between virulence on susceptible hosts and elicitation of non-host-plant defenses. Indeed, the need for tight regulation of effector expression has been noted previously in light of evidence suggesting that some effectors stimulate the hypersensitive response, or otherwise decrease bacterial fitness *in planta*, in a dose-dependent manner (51, 52). Given that nonspecific, abiotic factors introduce an element of randomness in the dispersal of *P. syringae* cells (53), those genotypes that impose negative feedback on HrpL expression may experience a positive fitness effect compared to the otherwise more virulent strains when populations are spread across a variety of plant hosts.

In support of this model, we present evidence to suggest that the function of the DC3000 T3SS is highly sensitive to an only modest increase in the concentration of HrpL. Graded constitutive expression of HrpL in excess of its native concentration was performed as a proxy for relief of negative feedback. In the absence of a target host plant cell, only a small subset of T3SS-associated proteins are secreted into culture medium (54), including the harpins HrpZ1 and HopP1 and the effector AvrPto1 (29). Comparing the relative abundances of these T3SS substrates between intracellular and secreted protein fractions, it is apparent that a 2-fold increase in HrpL copy number is sufficient to saturate T3SS activity. Whereas the HrpA1 pilus subunit is more rapidly exported in response to a HrpL-dependent increase in expression, the HrpZ1, HopP1, and AvrPto1 substrates accumulate inside the cell. A restriction on T3SS activity experienced specifically by substrates translocated through the pilus but not the pilus subunit itself might arise due to (i) a pilus-dependent rate-limiting translocation step or (ii) an inability to switch from pilus formation to substrate secretion. Given that the *Salmonella enterica* serovar Typhimurium T3SS needle channel is less than 3 nm in diameter (2), necessitating that effectors transverse it in a fully unfolded state, substrate saturation of the pilus is very plausible. Alternatively, an imbalance in the normal stoichiometry of T3SS proteins caused by HrpL overexpression may affect the dynamics of substrate switching. A conserved cytoplasmic sorting platform governs the hierarchical, chaperone-dependent loading of substrates at the base of the *S.* Typhimurium T3SS (55), and a conformational change in this region accompanies the switch from needle formation to effector secretion (2). How substrate switching is regulated by the T3SSs of plant pathogens has yet to be fully explored, although it has been hypothesized that the switch from pilus assembly to effector secretion is coupled to penetration of the host cell membrane (54). It is plausible that substrate overexpression, in particular, that of HrpA1, inhibits a concentration-dependent regulatory event required for harpin translocation. In support of this hypothesis, it has been noted that plasmid-mediated overexpression of *hrpA1* can inhibit the ability of *P. syringae* to elicit the hypersensitive response (45).

In either scenario, tight control of HrpL expression is theoretically advantageous. In the first case, the accumulation of surplus, nonsecreted substrates represents a futile metabolic cost. Given that a DC3000 cell can secrete on the order of 10^5^ HrpA1 and 10^4^ AvrPto1 molecules per hour (29), the burden of T3SS expression is sizeable. Indeed, ΔT3SS mutants have a growth advantage over wild-type cells (56). In the second scenario, the hierarchical dynamics of T3SS function depends on coordinated expression of the T3SS regulon and therefore on HrpL abundance. This is exemplified by the increase in the level of HrpA1 released into the cell supernatant when HrpL is modestly overexpressed. The presence of HrpA1 in this fraction can be attributed to mechanical shearing or depolymerization of the pilus or to complete secretion into the extracellular space. Although the relative levels of significance of these processes *in planta* are uncertain, the pilus protein is thought to be a general elicitor of plant immune defenses. The *hrpA* sequence displays signatures of positive selection for substitutions that enable escape from immune recognition (57). Clearly, negative feedback by HrpL is advantageous in the context of immune evasion.

A notable limitation of our study was that the significance of *NAC* by HrpL was explored predominantly *ex planta*. This requires several assumptions to be made, the principal being that fine control of HrpL is as relevant in the complex plant environment as is apparent here in culture medium, albeit one mimicking the apoplast. No fitness effect of HrpL misregulation was observed in our simplified model system for host infection. More elaborate plant assays, also performed on nonhosts, are required to validate this. Similarly, our interpretation of the T3SS activity data assumes that the rates of substrate expression and secretion observed are intrinsic rather than regulated. Instead, it is plausible that T3SS function is regulated differently *in planta* from in culture. However, several technical challenges currently limit the applicability of quantitative proteomics to complex *in planta* samples.

### Concluding remarks.

Recognizing negative autogenous control by HrpL advances our knowledge of the regulatory system controlling T3SS gene expression in DC3000. Not only is this mechanism of fundamental interest with regard to σ^54^-regulated transcription, but it also highlights the importance of exploring the complexity that underlies otherwise well-defined genetic networks. We also argue that negative feedback on HrpL expression has important implications for the ecology of the DC3000 pathovar. It will be of future interest to determine the extent to which *NAC* is conserved among *P. syringae* strains and other Hrp group 1 plant pathogens and whether there exists a correlation between the expression levels of T3SS components and different pathogenic strategies.

## MATERIALS AND METHODS

### General microbiology and molecular biology.

The bacterial strains and plasmids used in this study are described in Table S1 in the supplemental material. *E. coli* and DC3000 were grown in lysogeny broth (LB) at 37°C and 28°C, respectively. *hrp* gene expression in DC3000 was stimulated with *hrp*-inducing medium (HIM) (25) at 25°C. Plasmid cloning procedures, protein purifications, and markerless gene deletions in DC3000 are described in Text S1.

10.1128/mBio.02273-16.9 TABLE S1  Strains and plasmids used in this work. Download TABLE S1, DOCX file, 0.04 MB. Copyright © 2017 Waite et al. 2017 Waite et al. This is an open-access article distributed under the terms of the Creative Commons Attribution 4.0 International license.

10.1128/mBio.02273-16.10 TEXT S1  Supplementary methods. Download TEXT S1, DOCX file, 0.04 MB. Copyright © 2017 Waite et al. 2017 Waite et al. This is an open-access article distributed under the terms of the Creative Commons Attribution 4.0 International license.

### Assay of *in vivo* promoter activity.

Cell fluorescence derived from transcriptional fusion reporter plasmids was measured simultaneously with OD_600_ at the population level under microwell conditions using a FLUOstar fluorometer (BMG). GFP fluorescence (485-nm excitation [ex.]/520-nm ± 10-nm emission [em.]) and RFP fluorescence (584-nm ex./620-nm ± 10-nm em.) were detected using standard settings. Fluorescence per unit cell growth, blank corrected against growth medium autofluorescence, was measured across three biological replicate cultures at 20-min intervals over 20 h. Starting cell culture densities were normalized to an OD_600_ of 0.25 for DC3000 and of 0.05 for *E. coli*.

### Analysis of T3SS transcript expression by RNA sequencing.

DC3000 and Δ*hrpL* strains were grown in duplicate in LB medium for 16 h at 28°C before being washed in 10 mM MgCl_2_ and resuspended to an OD_600_ of 0.25 in 500 ml HIM. After 4 h of growth at 25°C, the cells were fixed with a 1/10 vol of 5% phenol–95% ethanol (vol/vol) and harvested by centrifugation. Adopting a 5′ end-selective methodology for analysis of the primary transcriptome (58), whole-cell RNA preparation, library quality control, next-generation sequencing, and read alignment experiments were performed commercially (Vertis Biotechnologie). The protocol used is detailed further in Text S1. Briefly, total RNA was treated to enrich for primary transcripts and fragmented (50 to 100 nt) enzymatically and the derivative cDNA libraries were sequenced via the use of an Illumina HiSeq 2000 platform, with parameters optimized for high sequencing depth values (stranded, 100 million reads, 50-nt read length). Reads were aligned to the DC3000 genome (GenBank accession no. AE016853) prior to sample library normalization and statistical analysis (CLC genomics workbench; CLC Bio). The alignment template was modified to include two additional features in the *hrpL* locus: a 50-nt 5′ section present in both strains [“*hrpL*(5′)”; nt 1542813 to 1542862] and a 100-nt section of the open reading frame (ORF) absent in the Δ*hrpL* strain due to markerless deletion [“*hrpL*(ORF)”; nt 1542987 to 1543086]. Gene expression values accounting for variation in sample libraries and gene length were inferred from the counts of uniquely mapped reads subject to quantile (59) and reads per kilobase per million (RPKM) (60) normalizations. Differential expression analysis was performed on normalized gene expression values (mean of two replicates) using Baggerley’s beta-binomial test (61) with a false discovery rate (FDR) threshold of 0.05 (62).

### Analysis of intracellular and secreted protein fractions.

Differential analysis of DC3000 protein fractions by multiple reaction monitoring–mass spectrometry (MRM-MS) was performed as described previously (29). HrpL and AvrPto1 proteins were doubly labeled at arginine and lysine residues *in vivo* for protein standard absolute quantification (PSAQ). Briefly, the coding sequences were PCR amplified and cloned into pET28b+ (Novagen) in frame with an N-terminal histidine tag. Gutnick minimal medium supplemented with 0.4% glucose, 10 mM NH_4_Cl, 1 mM heavy-labeled l-(^13^C_6_,^15^N_2_)-arginine, and l-(^13^C_6_,^15^N_2_)-lysine (Sigma-Aldrich) and 18 unlabeled amino acids (each at a 1 mM concentration) was used for protein expression in a modified Δ*argA* Δ*lysA* BL21 strain (63). Proteins were purified from inclusion bodies by nickel (Ni)-affinity chromatography in the presence of 7 M urea. Specific protein standard concentrations were calculated using a Bradford-based assay, correcting for impurities estimated by SDS-PAGE and fluorescent Sypro Ruby staining (Bio-Rad). DC3000 strains were grown in duplicate in LB medium for 16 h at 28°C before being washed in 10 mM MgCl_2_ and resuspended to an OD_600_ of 0.25 in triplicate 75-ml cultures in HIM (pH 6). After a further 24 h of growth at 25°C, the extracellular supernatant (“secreted”) and cell-associated (“intracellular”) protein fractions were subsequently separated by centrifugation of 30-ml samples. A mix of labeled HrpL and AvrPto1 standards was added to the supernatant fractions prior to concentration to 200 µl using an Ultra-15 centrifugal filter unit (Millipore) (molecular weight cutoff [MWCO], 15). The concentrated sample was incubated overnight at 37°C with 2 µg modified trypsin (Promega) and buffer T {100 mM Tris-HCl [pH 8], 50 mM NH_4_HCO_3_, 1 mM TCEP [*tris*(2-carboxyethyl)phosphine]}. Complete tryptic digestion was confirmed by SDS-PAGE prior to the addition of 2% formic acid. The corresponding cell pellet fractions were resuspended in 1 ml HIM supplemented with 7 M urea prior to disruption by sonication. A 20-µl sample of intracellular protein was subjected to tryptic digestion as described above. Tryptic peptides were analyzed using a QTrap 6500 mass spectrometer coupled to an ekspert nanoLC 400 liquid chromatography (LC) system (AB Sciex). Details of the working settings of the liquid chromatography-mass spectrometry (LC-MS) analysis are provided in Text S1. The peptide transitions analyzed by MRM are listed in Data set S1, adapting the previously optimized method (29). Data analysis was performed using Analyst software (AB Sciex). Relative levels of sample protein abundance were estimated using analyte peak intensities normalized for cell density at sampling.

### Exonuclease III footprinting.

Exonuclease III (ExoIII) footprinting was performed on variant P*hrpJ-hrpL* double-stranded DNA (dsDNA) promoter probes, labeled at the P*hrpJ* terminus with a 5′ cyanine (Cy3) dye molecule during PCR amplification using pTE103-P*hrpJ* and pTE103-P*hrpJ*(Δ35e) as the templates. Footprinting reactions (using 12-μl reaction mixtures) were performed in STA buffer (2.5 mM Tris-acetate [pH 8], 8 mM Mg-acetate, 10 mM KCl, 1 mM dithiothreitol, 3.5% [wt/vol] polyethylene glycol [PEG] 8000). The copurification of RNAP-HrpL and RNAP-HrpL_ΔR4_._2_ complexes is described in Text S1. IHF and σ^54^ protein samples were sourced from the laboratory collection and purified as previously described (15). Protein-DNA complexes were preincubated at 22°C for 10 min before the addition of 50 units ExoIII and proprietary buffer (Promega). Digestion was performed for 2 min before ExoIII was inactivated with 20 mM EDTA for 10 min at 70°C. Partially digested single-stranded DNA (ssDNA) products were run on an 8% urea footprinting gel. Cy3 fluorescence was detected using a FLA-5000 phosphorimager (Fujifilm) with a 488-nm excitation laser and a 532-nm emission filter.
