# Supplementary material for: Negative Autogenous Control of the Master Type III Secretion System Regulator HrpL in Pseudomonas syringae
Source: mBio. 2017 Jan 24;8(1):e02273-16. doi: 10.1128/mBio.02273-16 (PMC5263251; doi:10.1128/mBio.02273-16)
Supplement: TABLE S1 [file mbo002173158st1.docx]

**Negative autogenous control of the master type III secretion system regulator HrpL in *Pseudomonas syringae***

**Table S1 – Strains and plasmids used in this work.**

| **Strain** | **Genotype** | **Reference or Source** |
| --- | --- | --- |
| *E. coli* | | |
| MC1061 | araD139 Δ(araA-leu)7697 ΔlacX74 galK16 galE15(GalS) λ- e14- mcrA0 relA1 rpsL150(strR) spoT1 mcrB1 hsdR2 | (1) |
| s17 λpir | RPA-2, Tc::Mu-Km::Tn7(pir) | (2) |
| BL21(DE3) | F^–^, *dcm*, *ompT*, *hsdS*(r_B_^-^ m_B_^-^), *gal* λ(DE3) | Stratagene |
| BL21(DE3) Δ*lys*Δ*arg* | F^–^, *dcm*, *ompT*, *lys*, *arg*, *hsdS*(r_B_^-^ m_B_^-^), *gal* λ(DE3) | (3) |
| *P. syringae* | | |
| *DC3000* | Rif^r^ | (4) |
| Δ*hrpL* | DC3000(Δ*hrpL*::FRT) | (5) |
| Δ*hrpR* | DC3000(Δ*hrpR*::FRT) | This study |
| Δ*hrpS* | DC3000(Δ*hrpS*::FRT) | This study |
| Δ*hrpV* | DC3000(Δ*hrpV*::FRT) | This study |
| Δ*hrpG* | DC3000(Δ*hrpG*::FRT) | This study |
| Δ*hrpA1* | DC3000(Δ*hrpA1*::FRT) | This study |
|  | | |
| **Plasmid** | **Description** | **Reference** |
| pBBR1MCS4 | Medium-copy, broad-host range vector with pBBR1 replication origin. Amp^r^ | (6) |
| pSEVA521 | Low-copy, broad-host range vector with RK2 origin. Tet^r^ | (7) |
| pSEVA224 | Low-copy, broad-host range expression vector with *lacI^q^-P_TRC_* promoter and RK2 origin. Kan^r^ | (7) |
| pSEVA614 | Low-copy, broad-host range expression vector with *lacI^q^-P_TRC_* promoter and R6K origin. Gm^r^ | (7) |
| pET28b^+^ | Recombinant expression vector for *E. coli* BL21(DE3) with an IPTG-inducible T7/*lacO* promoter and 6-His tags. Kan^r^ | Novagen |
| pVS10 | *E. coli* *rpoA-rpoB-rpoC*[His6] and *rpoZ* genes under control of the T7 promoter. Amp^r^ | (8) |
| pTE103 | *In vitro* transcription template vector. Amp^r^ | (9) |
| pFLP2 | FLP flippase expression vector (cI_857_, Pλ*-flp*, *sacB*). Amp^r^ | (10) |
| pGEM-T | Multi-purpose TA-cloning vector. Amp^r^ | Promega |
| pGEM-T-KanFRT | pGEM-T derivative containing a *nptII*-FRT selection marker cassette. Amp^r^ Kan^r^ | (11) |
| pAPT-RS | pAPT110 expression vector containing a heterologous P*tac-hrpRS* operon. Kan^r^ | (12) |
| pSB4A3-P*hrpL*-*gfp* | Fluorescent reporter with *hrpL* promoter (-208 to +1) and synthetic RBS (BBa_B0030) fused to *gfp*(mut3b). Amp^r^ | (13) |
| pSB4A3-P*hrpL*-*rfp* | Fluorescent reporter with *hrpL* promoter (-208 to +1) and synthetic RBS (BBa_B0030) fused to *rfp*(mrfp1). Amp^r^ | (14) |
| pBBR1-P*hrpL*-*gfp* | pBBR1MCS4 derivative containing the P*hrpL-gfp* fusion from pSB4A3-P*hrpL*-*gfp* as an SphI-XbaI fragment. Amp^r^ | This study |
| pBBR1-P*hrpRS*-*gfp* | pBBR1-P*hrpL*-*gfp* derivative containing the *hrpRS* promoter (-1028 to +1) fused to synthetic RBS (BBa_B0030) and *gfp*(mut3b) as an SphI-BamHI fragment. Amp^r^ | This study |
| pBBR1-*rfp*-P*hrpL*-*gfp* | pBBR1-P*hrpL*-*gfp* derivative containing the RBS-*rfp* sequence from pSB4A3-P*hrpL*-*rfp* as a SphI fragment. Amp^r^ | This study |
| pBBR1-*rfp*-P*hrpL*(Δ35e)-g*fp* | pBBR1-*rfp*-P*hrpL*-*gfp* derivative carrying a triple nucleotide (GGA>AAC) substitution in the -35 element of the *hrpJ* hrp-box motif. Amp^r^ | This study |
| pBBR1-P*hrpL*(147)-*gfp* | pBBR1-P*hrpL*-*gfp* derivative containing a truncated fusion with a minimal 147bp P*hrpL* sequence as a SphI-XbaI fragment. Amp^r^ | This study |
| pSEVA224-*hrpL* | pSEVA224 derivative containing the DC3000 *hrpL* gene under the control of the inducible *lacI^q^-P_trc_* promoter and synthetic RBS BBa_B0030 as an EcoRI-XbaI fragment. Kan^r^ | This study |
| pSEVA224-*hrpL*_ΔR4.2_ | pSEVA224 derivative containing the 5’ 456bp of the DC3000 *hrpL* gene under the control of the inducible *lacI^q^-P_trc_* promoter and synthetic RBS BBa_B0030 as an EcoRI-XbaI fragment. Kan^r^ | This study |
| pSEVA-31-*hrpL* | pSEVA224-*hrpL* derivative with strong synthetic RBS BBa_B0031. Kan^r^ | This study |
| pSEVA-33-*hrpL* | pSEVA224-*hrpL* derivative with weak synthetic RBS BBa_B0031. Kan^r^ | This study |
| pBBR1-31-*hrpL* | pBBR1MCS4 derivative containing the DC3000 *hrpL* gene under the control of the constitutive BBa_J23105 promoter and strong synthetic RBS BBa_B0031 as an SphI-SacII fragment. Amp^r^ | This study |
| pBBR1-33-*hrpL* | pBBR1-31-*hrpL* derivative with weak synthetic RBS BBa_B0033. Amp^r^ | This study |
| pBBR1-31- *hrpL*_Δ4.2_ | pBBR1-31-*hrpL* derivative containing the truncated *hrpL*_Δ4.2_ sequence from pSEVA224-*hrpL*_ΔR4.2_. Amp^r^ | This study |
| pBBR1-33- *hrpL*_Δ4.2_ | pBBR1-33-*hrpL* derivative containing the truncated *hrpL*_Δ4.2_ sequence from pSEVA224-*hrpL*_ΔR4.2_. Amp^r^ | This study |
| pSEVA614-*hrpL* | pSEVA614 derivative containing the *hrpL* expression construct from the pSEVA-*hrpL* plasmid as an EcoRI-XbaI fragment. Gm^r^ | This study |
| pSEVA614-*hrpL*_Δ4.2_ | pSEVA614 derivative containing the *hrpL*_ΔR4.2_ expression construct from the pSEVA224-*hrpL*_ΔR4.2_ plasmid as an EcoRI-XbaI fragment. Gm^r^ | This study |
| pET28b-*hrpL*-myc | pET28b^+^ derivative carrying an untagged DC3000 *hrpL* gene and in-frame C-terminal Myc tag as an NcoI-HindIII. Kan^r^ | This study |
| pET28b-*hrpL*_ΔR4.2_-myc | pET28b^+^ derivative carrying an untagged 5’ 456bp of the DC3000 *hrpL* gene and in-frame C-terminal Myc tag as an NcoI-HindIII. Kan^r^ | This study |
| pTE103-P*hrpJ* | pTE103 derivative carrying the 208nt bi-directional P*hrpL-hrpJ* promoter region as an EcoRI-BamHI fragment. Amp^r^ | This study |
| pTE103-P*hrpJ*(Δ35e) | pTE103-P*hrpJ* derivative carrying a triple nucleotide (GGA>AAC) substitution in the -35 element of the *hrpJ* hrp-box motif. Amp^r^ | This study |
| pKOhrpL | pGEM-T derived allele exchange vector carrying the ∆*hrpL*::*nptII*FRT knockout allele. Amp^r^ Kan^r^ | (5) |
| pKOhrpR | pGEM-T derived allele exchange vector carrying the ∆*hrpR*::*nptII*FRT knockout allele. Amp^r^ Kan^r^ | This study |
| pKOhrpS | pGEM-T derived allele exchange vector carrying the ∆*hrpS*::*nptII*FRT knockout allele. Amp^r^ Kan^r^ | This study |
| pKOhrpV | pGEM-T derived allele exchange vector carrying the ∆*hrpV*::*nptII*FRT knockout allele. Amp^r^ Kan^r^ | This study |
| pKOhrpG | pGEM-T derived allele exchange vector carrying the ∆*hrpG*::*nptII*FRT knockout allele. Amp^r^ Kan^r^ | This study |
| pKOhrpA1 | pGEM-T derived allele exchange vector carrying the ∆*hrpA1*::*nptII*FRT knockout allele. Amp^r^ Kan^r^ | This study |

**Abbreviations**: Gm^r^, gentamycin resistant; Amp^r^, ampicillin resistant; Kan^r^, kanamycin resistant; Tet^r^, tetracycline resistant.

**References**

1. **Casadaban MJ**, **Cohen SN**. 1980. Analysis of gene control signals by DNA fusion and cloning in Escherichia coli. J Mol Biol **138**:179–207. doi:10.1016/0022-2836(80)90283-1.

2. **Simon R**, **Priefer U**, **Puhler A**. 1983. A Broad Host Range Mobilization System for Invivo Genetic-Engineering - Transposon Mutagenesis in Gram-Negative Bacteria. Bio-Technology **1**:784–791. doi:

3. **Matic I**, **Jaffray EG**, **Oxenham SK**, **Groves MJ**, **Barratt CL**, **Tauro S**, **Stanley-Wall NR**, **Hay RT**. 2011. Absolute SILAC-compatible expression strain allows Sumo-2 copy number determination in clinical samples. J Proteome Res **10**:4869–4875. doi:10.1021/pr2004715.

4. **Cuppels DA**. 1986. Generation and Characterization of Tn5 Insertion Mutations in Pseudomonas syringae pv. tomato. Appl Env Microbiol **51**:323–327. doi:

5. **Schumacher J**, **Waite CJ**, **Bennett MH**, **Perez MF**, **Shethi K**, **Buck M**. 2014. Differential secretome analysis of *Pseudomonas syringae* pv *tomato* using gel-free MS proteomics. Front Plant Sci **5**. doi:10.3389/fpls.2014.00242.

6. **Kovach ME**, **Elzer PH**, **Hill DS**, **Robertson GT**, **Farris MA**, **Roop RM**, **Peterson KM**. 1995. 4 New Derivatives of the Broad-Host-Range Cloning Vector Pbbr1mcs, Carrying Different Antibiotic-Resistance Cassettes. Gene **166**:175–176. doi:

7. **Silva-Rocha R**, **Martinez-Garcia E**, **Calles B**, **Chavarria M**, **Arce-Rodriguez A**, **de las Heras A**, **Paez-Espino AD**, **Durante-Rodriguez G**, **Kim J**, **Nikel PI**, **Platero R**, **de Lorenzo V**. 2013. The Standard European Vector Architecture (SEVA): a coherent platform for the analysis and deployment of complex prokaryotic phenotypes. Nucleic Acids Res **41**:D666–D675. doi:Doi 10.1093/Nar/Gks1119.

8. **Belogurov GA**, **Vassylyeva MN**, **Svetlov V**, **Klyuyev S**, **Grishin N V**, **Vassylyev DG**, **Artsimovitch I**. 2007. Structural Basis for Converting a General Transcription Factor into an Operon-Specific Virulence Regulator. Mol Cell **26**:117–129. doi:10.1016/j.molcel.2007.02.021.

9. **Elliott T**, **Geiduschek EP**. 1984. Defining a bacteriophage T4 late promoter: absence of a “-35” region. Cell **36**:211–219. doi:

10. **Hoang TT**, **Karkhoff-Schweizer RR**, **Kutchma AJ**, **Schweizer HP**. 1998. A broad-host-range Flp-FRT recombination system for site-specific excision of chromosomally-located DNA sequences: application for isolation of unmarked Pseudomonas aeruginosa mutants. Gene **212**:77–86. doi:

11. **Zumaquero A**, **Macho AP**, **Rufian JS**, **Beuzon CR**. 2010. Analysis of the Role of the Type III Effector Inventory of *Pseudomonas syringae* pv. *phaseolicola* 1448a in Interaction with the Plant. J Bacteriol **192**:4474–4488. doi:Doi 10.1128/Jb.00260-10.

12. **Jovanovic M**, **James EH**, **Burrows PC**, **Rego FGM**, **Buck M**, **Schumacher J**. 2011. Regulation of the co-evolved HrpR and HrpS AAA+ proteins required for *Pseudomonas syringae* pathogenicity. Nat Commun **2**:177. doi:10.1038/ncomms1177.

13. **Wang B**, **Kitney RI**, **Joly N**, **Buck M**. 2011. Engineering modular and orthogonal genetic logic gates for robust digital-like synthetic biology. Nat Commun **2**:508. doi:10.1038/ncomms1516.

14. **Wang BJ**, **Barahona M**, **Buck M**. 2013. A modular cell-based biosensor using engineered genetic logic circuits to detect and integrate multiple environmental signals. Biosens Bioelectron **40**:368–376. doi:DOI 10.1016/j.bios.2012.08.011.
