# Supplementary material for: Negative Autogenous Control of the Master Type III Secretion System Regulator HrpL in Pseudomonas syringae
Source: mBio. 2017 Jan 24;8(1):e02273-16. doi: 10.1128/mBio.02273-16 (PMC5263251; doi:10.1128/mBio.02273-16)
Supplement: TEXT S1 [file mbo002173158s1.docx]

**Negative autogenous control of the master type III secretion system regulator HrpL in *Pseudomonas syringae***

**Text S1 – Supplementary methods**

**Bacterial strains and growth conditions**

Bacterial strains used in this study are listed in Table S1. *Escherichia coli* and DC3000 derivative strains were grown in Lysogenic Broth (LB) medium at 37°C or 28°C, respectively. For induction of *hrp* gene expression, DC3000 cells grown in LB medium were washed twice in 10mM MgCl_2_ and re-suspended in a *hrp*-inducing medium (50mM sodium phosphate buffer pH 7, 1.7mM MgCl_2_, 1.7mM NaCl, 7.6mM (NH_4_)SO_4_, 10mM fructose) (1) and grown at 25°C. Where appropriate, media were supplemented with antibiotics at the following concentrations: ampicillin 100µg/mL; kanamycin 50µg/mL; gentamicin 10µg/mL; tetracycline 10µg/mL; rifampicin 80µg/mL.

**Plasmids and cloning procedures**

The plasmids used in this study are described in Table S1. To generate the pBBR1-P*hrpL*-*gfp* reporter plasmid, a transcriptional fusion consisting of a 208 nt *hrpL* promoter sequence, synthetic ribosome binding site (BBa_B0030, Registry of Standard Biological Parts (http://partsregistry.org)) and GFP (*mut3b*), was cloned from pSB4A3-P*hrpL*-*gfp* (2) into the broad-range plasmid pBBR1MCS4 as an SphI-XbaI fragment. An alternative 5’ primer was utilised to amplify a truncated variant of the transcriptional fusion, comprising only 147 nt of P*hrpL*, for generation of pBBR1-P*hrpL*(147)-*gfp*. The bi-directional and two-colour reporter pBBR1-*rfp*-P*hrpL*-*gfp* was subsequently derived from pBBR1-P*hrpL*-*gfp* with the insertion of BBa_B0030 and *mRFP1* cloned from pSB4A3-P*hrpL*-*gfp* (3) as a SphI fragment. The pBBR1-*rfp*-P*hrpL*(Δ35e)-g*fp* reporter was derived by site-directed mutagenesis of pBBR1-*rfp*-P*hrpL*-*gfp* using complementary oligonucleotides to both initiate inverse PCR and introduce a GGA>AAC substitution at the -35 element of the *hrpJ* hrp-box motif. pBBR1-P*hrpRS*-*gfp*, an alternative reporter consisting of an extended *hrpRS* promoter (-1028 to +1) fused to *gfp*(mut3b) was generated using the pBBR1-P*hrpL*-*gfp* plasmid as a template. The *hrpRS* promoter sequence was amplified from DC3000 chromosomal DNA as a SphI-BamHI template.

To generate the supercoiled DNA templates for the *in vitro* transcription assay, both the native 208nt P*hrpL* sequence and the Δ35e derivative were cloned into *in vitro* transcription template pTE103 as EcoRI-BamHI fragments.

The *hrpL* coding sequence was PCR amplified from purified DC3000 chromosomal DNA for cloning into expression plasmids. Identical primer pairs were used to clone *hrpL* as an EcoRI-XbaI fragment into the multiple cloning sites shared by pSEVA224 and pSEVA614. A series of synthetic RBSs were introduced by the 5’ primer to enable alternative expression levels of *hrpL* (BBa_B0030, high affinity, pSEVA224-*hrpL*; BBa_B0031, high affinity, pSEVA-31-*hrpL*; BBa_B0033, low affinity, pSEVA-33-*hrpL*). To generate stable vectors for *hrpL* expression during seedling infection, constructs comprising a constitutive promoter (BBa_J23105, Registry of Standard Biological Parts) and either the *hrpL* or *hrpL*_ΔR4.2_ coding sequences was cloned into pBBR1MCS4 as SphI-SacII fragments. pBBR1-31-*hrpL* and pBBR1-33-*hrpL* utilise the BBa_B0031 and BBa_B0033, respectively, for regulation of *hrpL* expression rate. An empty vector control was used as a comparison control in all complementation experiments.

A NcoI-HindIII fragment consisting of *hrpL* and a C-terminal Myc tag was cloned into pET28b^+^ for protein overexpression. For each expression plasmid, an alternative 3’ primer was utilised to amplify a truncated 456nt variant of *hrpL* for generation of a *hrpL*_Δ4.2_ derivative.

Where appropriate, plasmids were transformed into DC3000 using an electroporation protocol previously described for *P. aeruginosa* (4). Briefly, per aliquot of competent cells, 1 ml overnight culture grown in LB was centrifuged at 4000 x g and ambient temperature. The pellet was washed twice in 300 mM sucrose solution before being resuspended in 200 μl sucrose solution.

**Purification of RNAP-HrpL holoenzymes**

The RNAP core enzyme (6His-β’) and untagged HrpL derivatives were co-expressed in *E. coli* BL21 (DE3) from pVS10 and pET28b-*hrpL*-myc or pET28b-*hrpL*_ΔR4.2_-myc via induction with 0.5 mM isopropyl-β-D-thiogalactoside. Cell pellets were resuspended in buffer A (100mM Tris-HCl pH 7.4, 500mM NaCl, 5% glycerol and 0.1mM EDTA), disrupted by sonication and the resultant soluble fraction purified by metal affinity chromatography (His-Trap HP column, *GE Healthcare*) using a linear gradient of 0-1M imidazole (in buffer A). The desired protein fractions were dialysed twice against storage buffer (50 mM Tris-HCl pH 8, 100 mM NaCl, 20% glycerol (w/v), 1 mM dithiothreitol and 0.1 mM EDTA).

A Western blot was performed to confirm the presence of Myc-tagged HrpL and HrpL_ΔR4.2_ in co-purified samples. Protein was transferred from an SDS-PAGE-gel to an Immuno-Blot PVDF membrane (*Bio-Rad*) which was run for 60 mins at 100-200mA in a buffer of 10mM Tris, 100mM glycine and 10% (v/v) methanol. The membrane was blocked with 5% (w/v) Marvel milk in Tris-buffered saline solution (*Sigma*). The membrane was incubated with a monoclonal anti-Myc primary antibody (*Sigma*) at a 1:500 dilution followed by an HRP-conjugated anti-mouse secondary antibody (*Amdex*) at 1:5000 dilution. Bound antibody was stained with chemiluminescent ECL-Plus reagent (*GE Healthcare*) and visualised using a GelDoc XR System (*Bio-Rad*).

**Generation of markerless deletion mutants in DC3000**

Δ*hrpS*, Δ*hrpV*, Δ*hrpG* and Δ*hrpA1* knockout mutations were induced by allelic exchange as described previously (5, 6). Briefly, two ~700nt sequences corresponding to the 5’ and 3’ genomic flanking regions of the target gene were PCR amplified, fused by single-overlap extension PCR, utilising homologous overlaps in primer sequences, and cloned into pGEM-T (*Promega*), an effective suicide vector for DC3000. A curable kanamycin resistance cassette flanked by Flippase Recognition Targets (FRT-*nptII*-FRT) was cloned from the pGEM-T-KanFRT plasmid and ligated between each flanking region pair to yield the pKO series of allele exchange vectors.

Following double recombination in DC3000, confirmed by antibiotic selection and diagnostic PCR combining locus- and *nptII*-specific primers, Flippase-mediated curing of the *nptII* gene was induced using the pFLP2 plasmid (7), leaving a residual 111nt scar sequence, containing a single FRT site, in place of the target gene. Finally, FLP2 was removed via sacB-mediated counter-selection.

**Flow cytometry**

DC3000 cells carrying the pBBR1-P*hrpL*-*gfp* reporter plasmid were extracted from a fluorescence assay after 8 hours growth in *hrp*-inducing medium and diluted in fresh HIM to a density of 10^6^ CFU/ml. Flow cytometry was performed on an LSR Fortessa II cell analyser (*BD Biosciences*), which was washed with fresh HIM after each sample was analysed. 1000 events were detected for analyser calibration and initial population definition. The population of single cells belonging to a non-fluorescent DC3000 culture was electronically gated using forward and side light-scatter properties. 10000 cells of subsequent samples were analysed to obtain population fluorescence data in triplicate. Cells were excited with a 488nm laser and GFP fluorescence was detected using a 530/30 emission filter and 550LP mirror. Histograms of population fluorescence and other statistical parameters were generated using FlowJo 10 software (*FLOWJO, USA*).

### In vitro abortive transcription assay

Abortive small primed RNAs (spRNAs) were synthesised in 10μl reaction volumes containing: 200nM purified RNAP-HrpL/HrpL_Δ4.2_ holoenzyme or 160nM RNAP core enzyme (*Epicentre*), 20nM supercoiled promoter DNA (pTE103-P*hrpJ* or pTE103-P*hrpJ*(Δ35e)), 0.5mM dinucleotide initiating primer (CpA), 2mM UTP, and 0.2μCi/μl [α-32P]ATP (3000Ci/mmol) in Abortive Buffer [10 mM Tris-HCl, pH 7.5, 50 mM KCl, 10 mM MgCl_2_, 1 mM dithiothreitol and 0.1% w/v Triton X-100]. After 15 minutes at 25°C, transcription reactions were terminated with 10% (final) formamide and the products run on a 20% urea sequencing gel. An imaging plate was exposed to the gel for 30 minutes prior to signal detection using an FLA-5000 phosphorimager (*FujiFilm*). Densitometry of spRNA products was performed using AIDA Image Analyzer software (*Raytest*).

***Arabidopsis thaliana* seedling infection assays**

*Arabidopsis thaliana* Col-0 seedlings were grown on solid Murashige and Skoog (MS) medium. Seeds were vernalized for 2 days at 4°C prior to sterilisation in 70% ethanol and 50% sodium hypochloride before four rinses with sterile distilled water. Seedlings were grown in sets of sixteen at 22°C and 120 μmol photons m^‑2^ on sterile 120 x 120 mm culture plates. After 2 weeks, seedlings were flood inoculated with DC3000 strains, as described previously (8, 9). Cells were grown for 16 hours in LB at 28°C and washed twice in 10mM MgCl_2_ before resuspension in a solution containing 0.025% Silwet L-77. Plates were flooded in triplicate with 50ml cell suspensions at a density of 5x10^5^ CFU ml^-1^ and sealed with surgical tape (*Millipore*). Mock inoculations with 10mM MgCl_2_ solution were performed as a control. Bacterial populations were quantified at 0, 1, 2 and 3 days post infection (dpi) by serial dilution of CFU recovered from homogenised leaf tissue onto LB agar supplemented with Rifampicin and, where applicable, a second antibiotic. For each strain, CFU were measured for three technical replicate samples per time point, each consisting of three individual seedlings, and normalised for leaf sample wet weight. Leaf samples were homogenised in 500μl 10mM MgCl_2_ using a manual pestle. Disease symptoms on intact seedlings were logged after 3-4 dpi.

**Generation of cDNA libraries for RNA sequencing (*Vertis Biotechnologie*)**

Cell samples were treated with lysozyme for 15 minutes in order to extract total RNA which was then isolated using the mirVana microRNA isolation kit (*Ambion*). DNase was used to specifically remove contaminant DNA. Primary transcript enrichment was achieved by treating RNA samples with Terminator-5′-phosphate-dependent exonuclease (*Epicentre Biotechnologies*) to remove other processed RNAs. The primary RNA transcripts were fragmented enzymatically with RNaseIII to a length of 20-100nt before addition of poly(A) tails using poly(A) polymerase. 5' triphosphate structures were removed with RNA 5' polyphosphatase (*Epicentre*) before T4-RNA ligase was used to add an adapter onto the remaining 5’ monophosphate. First-strand cDNA synthesis by a Moloney Murine Leukemia Virus (M-MLV) reverse transcriptase was primed using a 3’ TrueSeq Antisense oligo(dT)-adapter primer (*Illumina*) complementary to the 3’ poly(A) tail. Second-strand synthesis was primed by the 5’ TrueSeq Sense primer, complementary to the 5’ RNA adapter. The resulting cDNA was PCR-amplified to about 15-20 ng/μl (10-11 thermocycles) using a high fidelity DNA polymerase and purified using the Agencourt AMPure XP kit (*Beckman Coulter Genomics*). cDNA was pooled in equimolar quantities and sequenced on an Illumina HiSeq 2000 machine. Prior to genome alignment, the output 50bp Illumina sequencing reads in Sanger fastq format were trimmed to remove adapter sequences and duplicate reads were filtered out.

**References**

1. **Huynh T V**, **Dahlbeck D**, **Staskawicz BJ**. 1989. Bacterial blight of soybean: regulation of a pathogen gene determining host cultivar specificity. Science **245**:1374–1377. doi:10.1126/science.2781284.

2. **Wang B**, **Kitney RI**, **Joly N**, **Buck M**. 2011. Engineering modular and orthogonal genetic logic gates for robust digital-like synthetic biology. Nat Commun **2**:508. doi:10.1038/ncomms1516.

3. **Wang BJ**, **Barahona M**, **Buck M**. 2013. A modular cell-based biosensor using engineered genetic logic circuits to detect and integrate multiple environmental signals. Biosens Bioelectron **40**:368–376. doi:DOI 10.1016/j.bios.2012.08.011.

4. **Choi KH**, **Kumar A**, **Schweizer HP**. 2006. A 10-min method for preparation of highly electrocompetent *Pseudomonas aeruginosa* cells: Application for DNA fragment transfer between chromosomes and plasmid transformation. J Microbiol Methods **64**:391–397. doi:DOI 10.1016/j.mimet.2005.06.001.

5. **Zumaquero A**, **Macho AP**, **Rufian JS**, **Beuzon CR**. 2010. Analysis of the Role of the Type III Effector Inventory of *Pseudomonas syringae* pv. *phaseolicola* 1448a in Interaction with the Plant. J Bacteriol **192**:4474–4488. doi:Doi 10.1128/Jb.00260-10.

6. **Schumacher J**, **Waite CJ**, **Bennett MH**, **Perez MF**, **Shethi K**, **Buck M**. 2014. Differential secretome analysis of *Pseudomonas syringae* pv *tomato* using gel-free MS proteomics. Front Plant Sci **5**. doi:10.3389/fpls.2014.00242.

7. **Hoang TT**, **Karkhoff-Schweizer RR**, **Kutchma AJ**, **Schweizer HP**. 1998. A broad-host-range Flp-FRT recombination system for site-specific excision of chromosomally-located DNA sequences: application for isolation of unmarked Pseudomonas aeruginosa mutants. Gene **212**:77–86. doi:

8. **Ishiga Y**, **Ishiga T**, **Uppalapati SR**, **Mysore KS**. 2011. *Arabidopsis* seedling flood-inoculation technique: a rapid and reliable assay for studying plant-bacterial interactions. Plant Methods **7**:32. doi:10.1186/1746-4811-7-32.

9. **Engl C**, **Waite CJ**, **McKenna JF**, **Bennett MH**, **Hamann T**, **Buck M**. 2014. Chp8, a diguanylate cyclase from *Pseudomonas syringae* pv. *tomato* DC3000, suppresses the pathogen-associated molecular pattern flagellin, increases extracellular polysaccharides, and promotes plant immune evasion. MBio **5**:e01168-14. doi:10.1128/mBio.01168-14.
